# Platelet activating factor receptor antagonists improve the efficacy of experimental chemo- and radiotherapy

**DOI:** 10.6061/clinics/2018/e792s

**Published:** 2018-09-26

**Authors:** Ildefonso Alves da Silva Junior, Luciana Nogueira de Sousa Andrade, Sonia Jancar, Roger Chammas

**Affiliations:** I Departamento de Imunologia, Instituto de Ciencias Biomedicas, Universidade de Sao Paulo, Laboratorio de Imunofarmacologia, Sao Paulo, SP, BR; II Laboratorio de Oncologia Experimental, Centro de Investigacao Translacional em Oncologia, Instituto do Cancer do Estado de Sao Paulo (ICESP), Hospital das Clinicas HCFMUSP, Faculdade de Medicina, Universidade de Sao Paulo, Sao Paulo, SP, BR

**Keywords:** Platelet-activating factor (PAF), PAF receptor (PAFR), PAFR antagonists, tumor repopulation, radiotherapy, Chemotherapy

## Abstract

Platelet activating factor is a lipid mediator of inflammation, and in recent decades, it has emerged as an important factor in tumor outcomes. Platelet activating factor acts by specific binding to its receptor, which is present in both tumor cells and cells that infiltrate tumors. Pro-tumorigenic effects of platelet activating factor receptor in tumors includes promotion of tumor cell proliferation, production of survival signals, migration of vascular cells and formation of new vessels and stimulation of dendritic cells and macrophages suppressor phenotype. In experimental models, blocking of platelet activating factor receptor reduced tumor growth and increased animal survival. During chemotherapy and radiotherapy, tumor cells that survive treatment undergo accelerated proliferation, a phenomenon known as tumor cell repopulation. Work from our group and others showed that these treatments induce overproduction of platelet activating factor-like molecules and increase expression of its receptor in tumor cells. In this scenario, antagonists of platelet activating factor markedly reduced tumor repopulation. Here, we note that combining chemo- and radiotherapy with platelet activating factor antagonists could be a promising strategy for cancer treatment.

## INTRODUCTION

Platelet-activating factor (PAF) was first described in the early 1970s by Benveniste et al. 1 as a soluble factor released by leukocytes from allergic patients upon stimulation with the allergen. Structural characterization of the PAF molecule was performed a few years later through the synthesis of compounds with similar physicochemical properties 2. Since these reports, studies have focused on the involvement of PAF in diverse conditions, including the role of PAF in tumor growth, which indicated the important role for this lipid mediator in tumor progression and carcinogenesis 3,4.

PAF production relies on inflammatory stimuli or stress, such as that induced by radiotherapy and chemotherapy 5-10. However, under physiologic conditions, a small and continuous amount of PAF is generated from cell membrane phospholipids by *de novo* synthesis, which is responsible for the functional regulation of plasma membranes 11,12. During inflammation, large amounts of PAF are generated, which occurs through the remodeling pathway, where alkyl-acyl-glycerophosphocholines (GPC) are converted to PAF via the concerted action of phospholipase A2 and PAF-acetyltransferases (LPCATs). In addition to the PAF generated by enzymatic processes, a wide range of oxidized phospholipids that bind to the PAF receptor (PAFR) are generated by oxidative stress 13,14. Because these phospholipids can activate downstream signaling cascades similar to native PAF, we will use the designation PAFR agonists for all these lipids.

The receptor that binds PAF is a GPCR (G-protein coupled receptor), cloned by Sugimoto et al. 15, and its activation induces different effects depending on the cell type. PAFR was initially described in macrophages, polymorphonuclear leukocytes, and endothelial cells, among others 2-4. This receptor is also expressed in some tumor cells, and PAFR agonists are generated in the tumor microenvironment, where they exert tumor-promoting effects that are dependent on the direct effect on tumor cells or cells from the tumor microenvironment.

In this review, we will first discuss the effects of PAF in tumor cells and then the PAF effects on cells from the tumor microenvironment, such as macrophages and endothelial cells. Finally, the effect of PAFR antagonists on cancer treatment and in tumor cell repopulation after radio- and chemotherapy will be addressed.

## PAFR AND TUMOR CELLS

The expression of PAFR is elevated in several human tumor lineages [e.g., Kaposi's sarcoma cells 16, the endometrial cancer cell line HEC-1A 17, epidermoid carcinoma (A431 cells) 18, the stomach cancer cell line JR-St 19, and N1E-115 neuroblastoma cells 20]. High amounts of PAFR transcripts 1 and 2 were found in human hepatocellular carcinoma 21 and gastric adenocarcinoma 22. In tumor cells, PAFR activation through G-proteins and tyrosine kinases is transduced to downstream pathways, including NFkB, MAPKs, AKT, PI3 kinase and Src 3,23. Together, these PAFR-activated pathways play a central role in oncogenic processes by inducing tumor cell proliferation. PAF has been reported to promote non-small cell lung cancer (NSCLC) progression and metastasis by initiating a forward feedback loop between PAFR and STAT3 24. PAFR activation also inhibits PTEN activity, leading to phosphorylation of the PI3K and ERK pathways that are critical signals for survival, proliferation and differentiation of tumor cells 25. The role of PAF in tumor cell survival, proliferation and migration was also shown in ovarian cancer. Aponte et al. 26 found increased levels of PAFR in serous ovarian tumors compared to mucinous and benign tumors. The authors showed that in serous ovarian cancer cells, PAF promotes cell proliferation and, at the molecular level, PAFR activation was accompanied by phosphorylation of EGFR, Src, FAK and paxillin. A few years later, EGF binding to the EGF receptor was shown to transactivate PAFR, leading to cPLA2 activation and PAF production in ovarian cancer cells 27. In another study, the same authors 28 verified that both the PAFR and EGFR signaling pathways promote tumor cell survival and migration in this tumor type and that the combined targeting of both receptors significantly reduced tumor growth and progression in nude mice. In primary oral squamous cell carcinoma (OSCC), the enzyme responsible for PAF synthesis, LPCAT1, is overexpressed compared to that in normal tissue, and its silencing decreased tumor cell proliferation and invasiveness 29, indicating that the PAF/PAFR axis is responsible for sustained prosurvival and proliferative signaling in malignant cells.

PAF also contributes to the malignant development of esophageal squamous cell carcinoma by stimulating PI3K/AKT activation 30. Blockade of the PAFR pathway inhibits tumor growth of breast cancer 25, prostate cancer 31, and Kaposi's sarcoma 32. These effects were associated with the inhibition of tumor angiogenesis 33. Bussolati and colleagues demonstrated that breast cancer cells (MCF7, T-47D, MDA-MB231) express PAFR and produce PAF in response to in vitro stimulation with VEGF, bFGF, HGF, TNFα and thrombin 34, indicating that signals produced in the tumor microenvironment can induce activation of the PAFR pathway in tumor cells. Furthermore, PAF can activate cancer cells, macrophages and endothelial cells to amplify PAF production and PAFR expression on their membranes in autocrine, endocrine, paracrine and juxtracrine interactions 35.

## PAFR AND THE TUMOR MICROENVIRONMENT

In solid tumors, neoplastic cells grow in the stroma, which is primarily composed of neoformed vessels, connective tissue (endothelial cells, fibroblasts and extracellular matrix) and several cell types recruited from the bloodstream, constituting the tumor inflammatory infiltrate (mainly macrophages and lymphocytes) 36. The inflammatory infiltrate can provide signals that inhibit or stimulate tumor growth 37. Previous studies from our group have linked the PAF/PAFR axis with suppression of tumor immunity. We found that two types of murine tumors, B16F10 melanoma and TC-1 carcinoma, showed significantly less growth in mice genetically deficient for PAFR (PAFRKO) than in wild type (WT) mice. This change occurred in parallel with the increased frequency of neutrophils and CD4^+^/CD8^+^ lymphocyte infiltration in the tumors implanted in PAFRKO, suggesting that PAFR may be involved in the recruitment of these cells into the tumor stroma 38. Macrophages undergo functional and phenotypic changes in response to signals from the tumor microenvironment. The role of macrophages in neoplasias is complex because macrophages can be reprogrammed towards anti- or protumoral phenotypes 39. M1 phenotype or "classically activated" macrophages have antitumor activity, and M2 macrophages have protumor effects 40. Moreover, when we analyzed the macrophages infiltrated in the tumors on day 15, the PAFKO mice had a higher frequency of M1-like macrophages (CD11c^+^; high iNOS; low arginase; low IL10) than WT mice, whereas those from WT mice presented the M2 phenotype marker (CD206^+^). Thus, in the absence of PAFR, the tumor infiltrating macrophages acquire an activated phenotype, which is associated with the reduced tumor growth observed in PAF KO animals 38, suggesting that PAFR activation reprograms macrophages towards the M2 phenotype in experimental tumors.

The PAF/PAFR axis can also suppress the immune response by inducing tolerogenic dendritic cells 41 and proliferation of myeloid-derived suppressor cells 42. In these cells, PAFR-mediated suppression involves IL10 and prostaglandin production 43. PAFR-dependent immunosuppression was suggested to occur via activation of regulatory T cells (Tregs) 44-46.

PAFR signaling can also promote modifications in the tumor microenvironment by inducing angiogenesis through activation of matrix remodeling metalloproteases (MMPs) and stimulation of vascular endothelial growth factor (VEGF) production via NFkB activation 47-50. In vivo, a PAF receptor antagonist inhibited metastasis of human melanoma cells implanted in nude mice 44. PAF also stimulated CREB-dependent expression and activation of MMP-2 and vascular endothelial cell invasion and migration in melanoma 51. Through activation of the IL-6/STAT3 axis, PAFR induced epithelial-mesenchymal transition in NSCLC cells 24. In addition, PAF has been suggested to affect tumor cell (colon carcinoma and melanoma) adhesiveness to endothelial cells 52.

These results indicate that PAFR signaling may have direct and indirect effects in promoting cancer progression and growth and that interfering with PAFR pathways may control tumor growth.

## PAFR AND TUMOR REPOPULATION AFTER CHEMO- AND RADIOTHERAPY

Based on the prosurvival and proliferative effects of PAF in tumor cells described here, it is likely that the signaling pathways evoked by PAFR might protect tumor cells during chemo- and radiotherapy. Although PAF was shown to enhance cell death induced by chemotherapy in an epidermal carcinoma model transduced with PAFR 7, the opposite effect has been reported by others. In 2006, Heon Seo et al. 53 observed that PAF led to an increase in the protein levels of the antiapoptotic proteins Bcl-2 and Bcl-xL in B16F10 melanoma cells and that costimulation of tumor cells with PAF and the chemotherapeutic drug etoposide protected melanoma cells from etoposide-induced cell death in an NFkB-dependent manner.

Several years later, studies from Onuchic et al. 54 showed that in PAFR-expressing human melanoma cells (SKMel37), cisplatin treatment increased PAFR expression in tumor cells. Treatment with exogenous PAF protected SKMel37 cells from cisplatin-induced cell death. Moreover, systemic treatment with a combination of cisplatin and the PAF-R antagonist WEB2086 substantially attenuated the growth of SKMel37 tumor xenografts in nude mice 54, suggesting that this signaling cascade might impair chemotherapeutic efficacy in melanoma. This effect was clearly demonstrated by Sahu et al. 55 in B16F10 melanoma-bearing mice treated with stable PAF (cPAF). Treatment of animals with cPAF caused a significant increase in tumor growth compared to that of the control group (vehicle only). In fact, cPAF abrogated the inhibitory effect of etoposide on tumor growth, highlighting the prosurvival role of PAF in some cancers.

In an ovarian cancer model, the same chemotherapeutic agent, cisplatin, led to an increase in PAFR mRNA and protein levels, which was mediated by NFkB and HIF-1α. Silencing of PAFR with siRNA or with a PAFR antagonist increased cisplatin-induced cell death in human ovarian carcinoma cells, indicating that PAF/PAFR might be involved in tumor cell survival after genotoxic stress. Using a SKOV-3-luciferase xenograft model, the authors also showed that the combined treatment with cisplatin and a PAFR antagonist (ginkgolide B) inhibited tumor progression 56, reinforcing the hypothesis that the PAF/PAFR axis is involved in tumor survival and that its blockade impairs tumor progression.

However, it has been demonstrated in melanoma that chemotherapy can generate PAFR agonists, which might reduce chemotherapeutic efficacy in this tumor type. The chemotherapeutic agents etoposide, dacarbazine, and cisplatin generate PAF-like molecules in a time- and dose-dependent manner in B16F10 cells. Furthermore, in a model of dual injection of B16F10 cells in WT mice, intratumoral treatment with melphalan or etoposide in one tumor augmented the growth of untreated tumors in a PAFR-dependent manner 57.

Following chemo- and radiotherapy, stressed and dying cancer cells release a variety of substances, including reactive oxygen as well as nitrogen species along with cytokines, such as IL-6, IL-8, and TNF-α, which can activate PAFR agonist production in the tumor microenvironment 14,58,59. This tumoral tissue response to genotoxic therapies represents an indirect pathway to tumor survival through PAFR activation.

Under these conditions, surviving tumor cells undergo cell proliferation, a phenomenon known as tumor cell repopulation. In a recent Opinion article, Ichim and Tait (2016) 60 discussed the intrinsic mechanisms that could explain the unwanted effects of therapies based on cell death. They noted an early observation made by Revesz in 1956 61 that mixing viable tumor cells with dying cells increases tumor growth. Since then, the molecular mechanisms behind this phenomenon have been investigated, and in 2011, Huang et al. 62 showed that in tumor cells undergoing apoptosis after radiotherapy, activated caspase3 activates cPLA2, leading to PGE2 synthesis and tumor repopulation.

Several groups have demonstrated that the so-called cancer stem cells (CSCs) are responsible for tumor repopulation. Evidence from preclinical studies demonstrated that during chemotherapy, there is an increase in the frequency of a subpopulation of quiescent or CSCs that re-enter the cell cycle, contributing to tumor repopulation 63-65. The activation of these slow cycling cells is caused by the release of different factors from dying cells, such as PGE2. An elegant study by Kurtova et al. 66 showed that blockade of PGE2 abrogates tumor repopulation in response to chemotherapy in bladder urothelial carcinoma xenografts. PGE2 release by tumor cells in response to gemcitabine and cisplatin recruited CSCs to proliferate and repopulate the damaged tissue as observed in normal wound healing.

Interestingly, in 2012, we demonstrated that PAF is at least partially responsible for this mitogenic effect of dying cells. In this study, we observed that the injection of a subtumorigenic dose of murine melanoma cells admixed with apoptotic cells promoted tumor engraftment and growth, and this effect was dependent on PAFR activation 67. In addition, we also found that increased PGE_2_ secretion by irradiated carcinoma cells and PAFR antagonists prevented PGE2 production and COX2 expression 68. Although both PAF and PGE2 induce cell proliferation and survival, PGE_2_ production was dependent on PAF in our studies. This finding can be explained by the fact that the PAFR pathway is often associated with arachidonic acid release 12, which can be converted to prostaglandins through specific enzymes.

In fact, the COX2/PGE2 axis is involved in resistance and the stemness phenotype in many malignancies 69. Based on the fact that the CSC phenotype is dynamic., i.e., a single tumor cell can acquire stem cell properties under certain circumstances, we speculate that the stem cell-enhancing activity of PGE2 might be downstream of PAF. However, it is unclear whether PAF can directly induce the stemness phenotype or CSC maintenance in response to genotoxic treatment, contributing to tumor repopulation and treatment failure.

In addition, stressed and dying cancer cells can also release pro-oxidative stressors that can act directly on GPC to produce oxidized GPC (ox-GPC), which is a potent PAFR ligand 7,70-73. The production of these “PAF-like” molecules is not under the controlled enzymatic process for PAF production. Similar to native PAF, these ox-GPCs can increase the proliferation of remaining tumor cells 9,10, contributing to tumor repopulation.

Recently, our group reported data that support the concept that the presence of PAFR ligands in the tumor microenvironment is a possible mechanism underlying tumor repopulation following chemo- and radiotherapy. Working with human and mouse cancer-derived cell lines, we first showed that tumor cells produce PAFR ligands after undergoing gamma irradiation. The irradiated cells co-cultured with live tumor cells exerted a *feeder* effect that significantly increased tumor cell proliferation, and this effect was markedly reduced by PAFR antagonists added to the co-cultures. In addition, when PAFR antagonists were added before irradiation, we found a significant decrease in tumor cell viability, suggesting that PAFR ligands protect tumor cells from death induced by radiotherapy 68. A similar effect was observed in experiments designed to simulate the repopulation phenomenon. Live TC-1 carcinoma cells were injected subcutaneously into mice together with irradiated TC-1 cells. These tumors grew significantly larger than those that were not mixed with irradiated cells. This effect was prevented in mice that were treated with a PAFR antagonist. Further experiments using a human oral carcinoma cell line that does not express PAFR (KBM) and the same cell line transfected with PAFR (KBP) confirmed these findings. PAFR^+^ and PAFR^–^ tumor cells admixed with irradiated tumor cells were injected subcutaneously in RAGKO mice, and tumor growth was monitored for 30 days. PAFR^+^ tumors became significantly larger and more vascularized than PAFR^-^ tumors. Repopulation in PAFR^+^ tumors was accompanied by an increase in protumoral macrophage (CD206+) infiltration 9. These results suggest that during radiotherapy, tumor cells that survive treatment will undergo accelerated growth, which is dependent on PAFR activation in tumor cells as well as tumor macrophages that acquire an activated phenotype.

In prostate cancer, PAFR was overexpressed in response to irradiation, and the PAFR antagonist ginkgolide B reduced cell viability. The radiosensitizing effect of this antagonist was observed in xenograft tumors, and this effect was shown to be mediated by the interaction between PAFR and beclin-1, leading to inactivation of autophagy 8.

Based on these studies, we propose that the PAF/PAFR axis may constitute an important route for acquired resistance after chemo- and radiotherapy in a direct or indirect manner, as highlighted previously 74. The combined use of PAFR antagonists with radio- or chemotherapeutic agents may be a promising strategy against cancer, increasing the radio- and chemotherapeutic effectiveness.

Treatments based on cell death trigger homeostatic mechanisms within tissues that favor the survival of surrounding cells. Tumor cell repopulation following chemo- and radiotherapy is the major drawback of these treatments. We have discussed here the mechanisms of treatment failure focusing on the PAF/PAFR axis. During these therapies, cell stress and oxidative processes generate a wide range of oxidized phospholipids that bind to PAFR. Our group has shown that blocking PAFR with selective antagonists was very effective in reducing tumor cell repopulation following chemo- and radiotherapy. Thus, PAFR antagonists could improve the efficacy of radiotherapy through inhibition of tumor repopulation. The association of radiotherapy with PAFR antagonists could be a novel and efficient therapeutic alternative in cancer treatment.

## AUTHOR CONTRIBUTIONS

Silva Junior IA and Andrade LN wrote the manuscript. Jancar S wrote the manuscript and participated in the conception and review of the study. Chammas R. participated in the conception and review of the study.
